# Biotagging method for animal identification using dissolvable microneedle arrays prepared by customisable moulds

**DOI:** 10.1038/s41598-023-50343-6

**Published:** 2023-12-21

**Authors:** Jongho Park, Beomjoon Kim

**Affiliations:** Institute of Industrial Science, The University of Tokyo, 4-6-1 Komaba, Meguro-Ku, Tokyo, 153-8505 Japan

**Keywords:** Biomedical engineering, Animal biotechnology

## Abstract

Properly handling animals and understanding their habits are crucial to establish a society where humans and animals coexist. Thus, identifying individual animals, including their possessions, and adequately managing each animal is necessary. Although several conventional identification methods exist, such as the use of ear punch, tattoos, and radio frequency (RF) chips, they require several processes and external apparatus. In this study, we proposed a new biotagging method using a microneedle array for animal identification. Our approach uses dissolvable microneedle arrays as a single patch to deliver dyes directly into the skin layer. Additionally, we developed a new fabrication method for customised female moulds to realise microneedle array patches (MAPs) with patterns of different characters and number. The characteristics and feasibility of the patterned MAPs were confirmed through basic evaluations and animal experiments. Moreover, we confirmed that patterns formed from biotagging using the developed patterned MAPs lasted over one month with clear readability. Finally, we confirmed that our patterned MAPs successfully realised biotagging on rat skin with the designated patterns including characters and number patterns. The proposed method is expected to enable minimally invasive tagging without external equipment or complex processes. In addition, the developed method could be used to embed various tags into the skin of animals and humans in the future.

## Introduction

The Animal Welfare Act (AWA) is a federal law that was enacted in 1966 in the United States. It regulates animal treatment in research, exhibitions, and transportation^1^. The primary objective of AWA is to prevent cruelty and suffering in animals and ensure humane treatment. Under AWA, animal welfare standards are established and applied to research facilities, exhibitors, and transporters. The law requires these facilities to provide animals with adequate housing, food, water, and veterinary care. In addition, a framework for enforcing these standards, including regular inspections of facilities and penalties for those found violating the law, has been established^2^.

Other countries have similar laws and regulations to protect animal welfare. For example, in the European Union (EU), animal welfare laws are implemented through the "European Convention for the Protection of Animals Kept for Farming Purposes" and the "Directive on the Protection of Animals Used for Scientific Purposes"^3,4^. In the United Kingdom, the Animal Welfare Act of 2006 provides a framework for protecting animal welfare and sets standards for animal housing, care, and treatment^5^. In other countries, such as Japan and South Korea, animal welfare acts focus on animal health and regulation of animal-related industries, such as livestock production and laboratory animal use^6,7^. Thus, it is well understood that animal management and its system are highly relevant in treating animals properly under the related laws and regulations described above.

Animal tagging, a useful method for identifying individual animals, plays an important role in animal identification and management. Animal tagging involves attaching a tag, device, or marker to an animal for identification or tracking purposes^8^. The purpose of animal tagging can vary, but it is commonly used for monitoring animal populations, tracking the movements of animals, and for research purposes^9^. Such animals include wildlife, industrial animals (such as livestock), and experimental animals. Thus, animal tagging has several advantages, such as providing a permanent identifier for the animal, allowing for easy tracking and monitoring, improving management, and conserving wildlife populations.

Various types of animal tags exist, including physical tags attached to or implanted inside the animal's body and physical markings such as ear punching, tattooing, and toe clipping. Among them, physical tags are typically used and are widely applied to industrial animals. Examples include ear tags^10^ and passive or active radio frequency identification (RFID) tags for livestock^11^. Physical tags are usually made of durable materials such as metals or plastics and can be easily seen or identified. Although ear tagging is a simple and rapid method, tags can be damaged or lost, resulting in inflammation and infection at the application sites. RFID tags embedded under the animal’s skin use radio waves to transmit information about the animal and can be read by a scanner or other electronic devices. Tagging chips are permanent and reliable; however, they require specialised equipment and an invasive implantation process. Additionally, collar tagging can be applied to household animals by attaching a collar worn around the animal’s neck. Collar tagging is widely used for pets such as dogs and cats because of its simplicity and convenience, even though a risk of damage or loss still remains owing to the behaviour of pets.

Physical markings can be used for small groups of animals, such as animals for experiments or pets. The most common methods include ear punching^12^ and tattooing^13^. They require a small tool to mark the body of the animals directly. Both methods produce permanent markings on the animal’s skin. In tattooing, unique number patterns can be included for straightforward identification. Overall, conventional tagging methods have several advantages, and they have been well established and used for a long time in various fields. However, each tagging method has disadvantages or limitations in that it requires additional equipment and is accompanied by invasive processes that can occasionally stress the animals. In addition, veterinarians and skilled personnel are required to perform surgical procedures to implant tags or microchips, which are costly. Therefore, new tagging methods or materials are required to address these issues.

Microneedle (MN) technology can be used as a solution to these problems. It is a relatively new and innovative approach, particularly for drug delivery and medical treatment. This technology involves the use of small (usually less than 1 mm in length) needles to penetrate the skin and deliver drugs, vaccines, or other pharmaceutical substances into the body^14^. Several types of MNs have been developed and studied. These include solid, coated, dissolvable, porous, and hydrogel types^15^. Each type has different characteristics and mechanisms of drug delivery or extraction of biofluids. A microneedle array patch (MAP) is a type of patch that has several MNs in a matrix array. As the patch is thin and flexible, MAP can be applied to the skin uniformly and directly, resulting in the release of drug material into the targeted skin layers, epidermis, or dermis, with penetration through the outermost barrier layer, the stratum corneum^15,16^.

MAP offers several advantages over traditional drug delivery methods, including reduced pain and minimal invasiveness, improved drug delivery efficiency, and increased usability and stability^16–19^. Moreover, this technique has great potential for mass production at a lower cost, making it more accessible and affordable to patients and medical staff. In addition to drug delivery, MAP can be used for painless and non-invasive monitoring of various biomarkers, such as glucose^20^, antibodies from immune responses^21^, proteins^22^, and hormones^23^. Recently, various applications using MAPs, such as improving iontophoresis to enhance transdermal molecular transport^24^ and medical tattoos using MAPs^25^, have been demonstrated. However, their implementation and application are still limited mainly for drug delivery or biosensing-related research.

**Figure 1:**
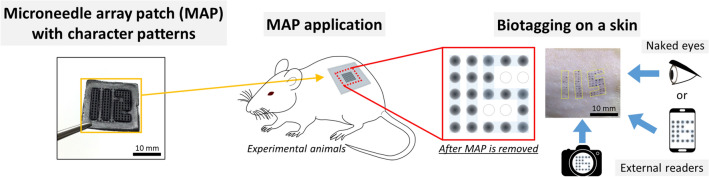
Schematic diagram of the developed MAP based biotagging technology.

In this study, we propose a new animal identification method using a dissolvable MAP that is easy to use and minimally invasive and does not require any marking equipment. We developed an improved micromoulding technique to achieve a MAP that has customised patterns without redesigning a master mould. Thus, we achieved simple and easy fabrication of female moulds that have various character patterns by using only one master mould as well as novel filling technique. We used tattoo inks to make visible marks on the skin by applying the MAP with an ink solution. Moreover, we simultaneously used each MN in the MN array as a dot matrix pattern to express specific characters and numbers for identification (Fig. 1). Briefly, dissolvable MNs were fabricated by mixing a base material, hyaluronic acid, and a black tattoo ink solution. Subsequently, the patterned MAP was prepared using a custom-designed female mould fabricated using our unique micromoulding technology. Finally, the fabricated MAPs were evaluated by experimenting on animals. The results confirmed the identification patterns on the skin as well as the duration of the MAP-derived patterns.

## Results

### Fabrication and basic evaluation of fabricated MAPs

In this study, we fabricated and used two types of female moulds to prepare MAPs: one was a base mould without patterns. The other was the mould for the patterned MAPs (Supplementary Information Fig. S1).

**Figure 2:**
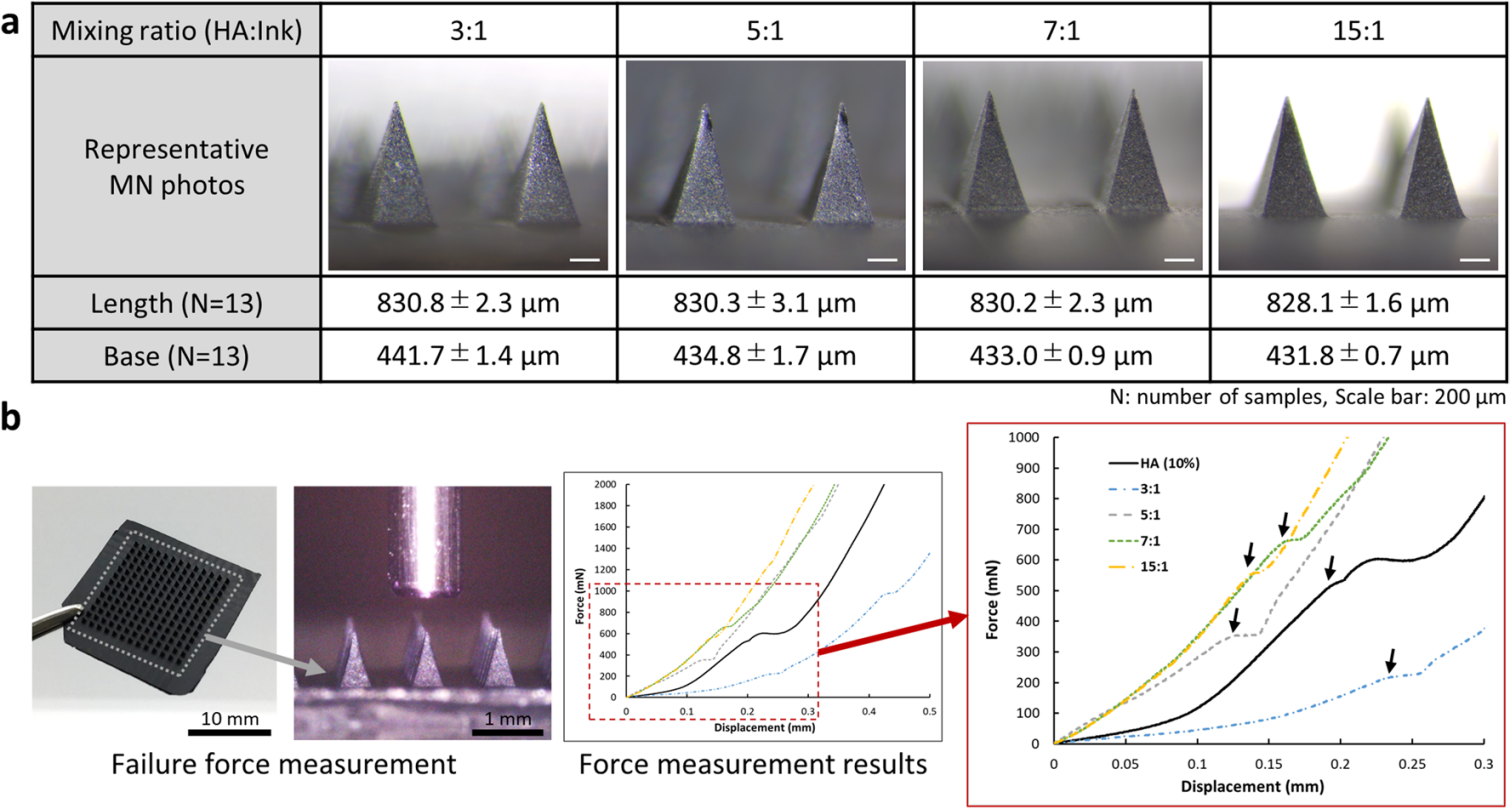
Fabrication and failure force evaluation of MAPs using mixed solutions at different ratios. (**a**) Fabrication of MAPs with different mixing ratios. (**b**) Evaluation of mechanical strength of single MN by compression tests.

First, we fabricated the MAPs using a mixed solution of hyaluronic acids and tattoo ink. We evaluated the feasibility of each mixing ratio and the formation of MNs in the elaborated MAPs. Here, base female moulds with no internal patterns were used for basic evaluations of fabricated MAPs. Figure 2a shows the results for different mixing ratios. Among the results obtained with all mixing ratios, we obtained fragmented and cracked MAP with a 1:1 mixing ratio. Carbon black could be considered one of the constituents of tattoo ink that causes an incomplete and cracked internal structure of the MAP^26,27^. Except for the 1:1 mixing ratio, all MAPs with other ratios were successfully fabricated with intact MN structures. Regarding the dimension of MNs for each step, MN lengths of a metal mould, a PDMS female mould, and fabricated MNs measured 872.2 ± 1.6, 858.0 ± 1.2, and 829.8 ± 2.3 μm, respectively (Fig. 2a, Supplementary Information Fig. S2). Approximately 1.6–3.2% shrinkage was observed at the midpoints of processes, including the curing process of PDMS mould fabrication and the drying process of MAPs. Similarly, the base lengths of the MNs decreased by approximately 1.6 and 5.3% during the female mould fabrication and drying processes, respectively (Fig. 2a, Supplementary Information Fig. S2).

Subsequently, the failure force of the MNs in each MAP was evaluated using a compression test (Fig. 2b). In particular, we measured the forces at which the inflexion points were observed in the force–displacement graphs (red rectangles). The average forces measured with each mixing ratio, 3, 5, 7, and 15:1, were 146.2 ± 13.2, 302.4 ± 20.7, 605.3 ± 31.7, and 603 ± 23.4 mN, respectively. Considering the mechanical strengths from previous studies, we considered that the fabricated MNs with all mixing ratios had sufficient mechanical strength (56 mN) to penetrate the skin layer^28^. In addition, our MNs exhibited much higher mechanical strength than hyaluronic acid-based MNs^29^. At the same time, we observed MNs with mixing ratios of 3:1 and 5:1 have lower mechanical strength than the control group, MNs without ink. On the contrary, MNs with mixing ratio of 7:1 as well as 15:1 had higher mechanical strength than the control group. From the results, we considered that different mechanical strengths were achieved from the different mixing ratio of hyaluronic acid and tattoo ink. We attribute the constituents in a tattoo ink, such as carbon black pigment and glycerine, to changes in mechanical strength depending on a mixing ratio. From the results, we finally chose a mixing ratio of 7:1 because the MNs have the highest mechanical strength and more ink volume than those with other mixing ratios.

### Fabrication of patterned female mould

We fabricated female moulds that were used for MAPs with specific patterns. Before fabrication, we designed patterns, including letter characters, numbers, and symbols so that they could be fabricated using patterned moulds and expressed in one MAP for identification.

**Figure 3:**
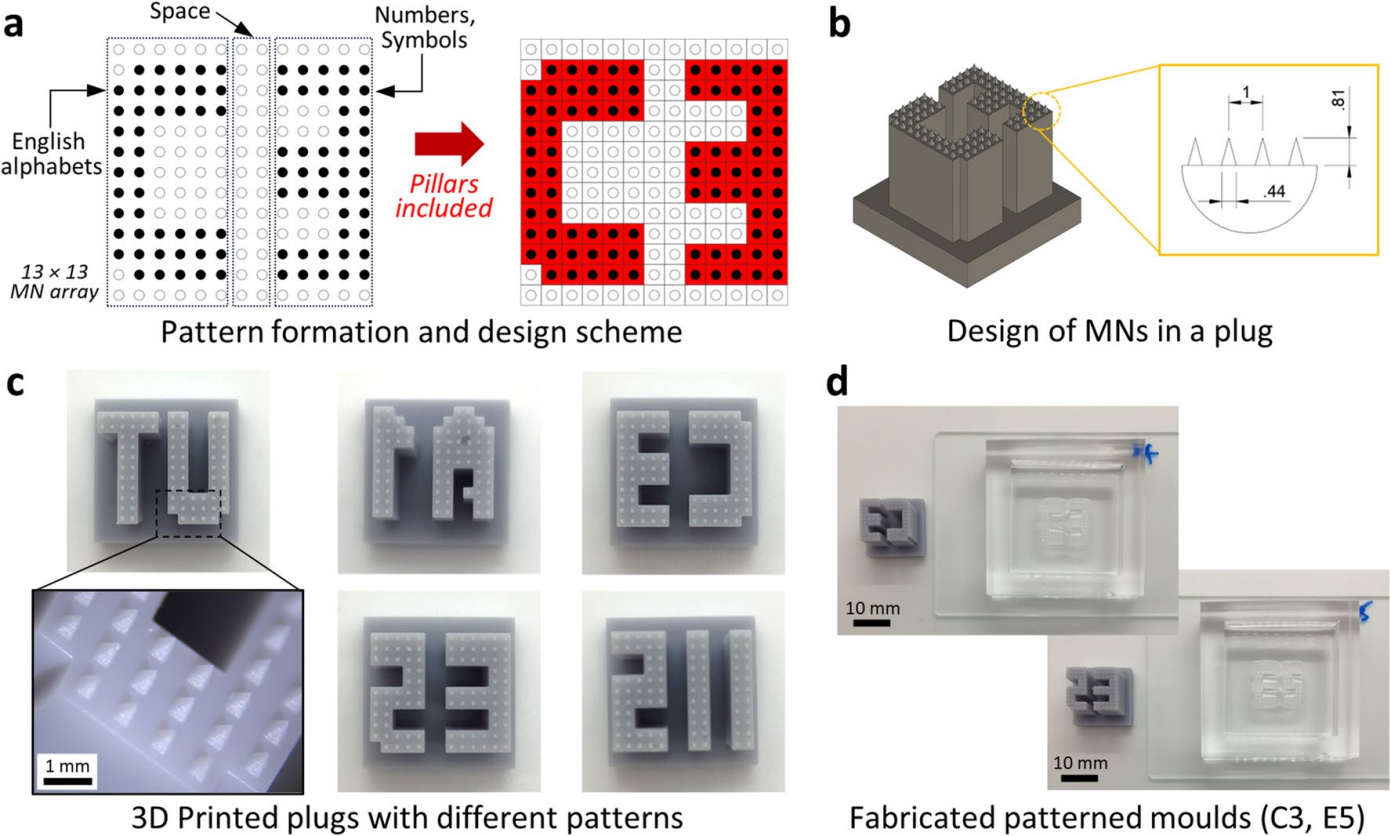
Design and fabrication of pattern plugs, with fabricated patterned female moulds. (**a**) Design of patterns for pattern plugs. (**b**) Designed dimensions for pattern plugs (all dimensions are in millimetres). (**c**) Fabricated pattern plugs with 5 different mixed patterns. (**d**) Representative fabricated patterned female moulds.

First, we set one MN, the inserted part of a MN during penetration, in the MN array as one dot of biotagging patterns. Thus, 169 dots, the same number as the cavities in the female base mould, existed in one MAP because the basic array of MNs was designed with a 13 × 13 size. In addition, we divided the entire array of 13 × 13 into three parts, including 6 × 13, 2 × 13, and 5 × 13, to express letters, spaces, numbers, and symbols, respectively (Fig. 3a). Then, one row at the top and bottom was blanked, considering the entire longitudinal length of the patterns. Thus, one MAP could express one English letter and number or symbol. The patterns can be recognised by imaginary outlines that are made up of multiple dot patterns. In addition, various letters or symbols can be expressed by adjusting the composition of MN array and number of MNs.

Inside the pattern, the spaces between each dot, both vertically and horizontally, were designed to be the same as the centre-to-centre distance of the MNs in the female base moulds. In addition, we set a virtual square block surrounding each dot pattern to realise the pillar structures of each patterned plug as a support for holding the plug shapes. These square blocks and dot patterns are indicated by red rectangles in Fig. 3a. Finally, we designed various patterns for the patterned plugs, considering the dimensions of the female base mould, processability, and readability. They included 26 English letter character patterns, 10 number patterns, 7 symbol patterns, one three-letter combination, and Chinese character patterns presenting the authors’ affiliations (Supplementary Information Fig. S3).

The detailed structures of the patterned plugs were designed to have pillars with pyramidal-shaped tips at the end (Fig. 3b). These pyramidal shapes can be used for positioning and filling cavities that should be left open in the base mould when fabricating the patterned moulds. In addition, the pillar structures support pyramidal tips, as described above, and secure the space for introducing a diluted PDMS mixture to fill unnecessary cavities. For dimensions, as described earlier in the Materials and Method section, pyramidal shapes at the tip of plugs were designed as 810 and 440 μm for the length and the base, respectively, so that all tips of pattern parts can fit in the cavities of a base mould. In this study, patterned plugs were fabricated with five different combinations that include the combination of a letter and number, and abbreviation letters of authors’ affiliations: A1, C3, E5, UT, and IIS.

Figure 3c shows plugs fabricated with different patterns. Pyramidal and supporting pillars were fabricated, as shown in the magnified inset. The pyramidal tips had a length of 600.7 ± 25.4 μm and a base length of 365.7 ± 1.7 μm. The pillar structures had designed lengths of 10 mm and 9.9 mm for the fabricated plugs. The vertical length was shorter than the designed value. In this regard, the dimensional changes might be caused by the time of exposure during 3D printing and the printing mechanism. The 3D printer created patterns layer by layer by utilizing UV exposure to the UV-curable resin. The exposure time for each layer significantly affected the vertical resolution and the resultant dimensions. In particular, increasing the exposure time and adjusting the designed dimensions might solve this issue. Although vertical lengths of pyramidal tips were confirmed to be smaller than those designed, the centre-to-centre distance, which is the most important for assembling a base mould and a pattern plug, was well matched: 994.5 ± 0.6 μm for a female base mould and 993.7 ± 0.9 μm for a fabricated pattern plug.

Finally, we confirmed that the fabricated patterned plugs fit well inside the cavities of the base mould, with uniform contact with the surfaces. Moreover, unnecessary cavities were successfully filled with masking cavities for patterns (Fig. 3d). We observed that the diluted PDMS completely merged with the base mould after curing and removing the plugs. The patterned moulds were cleaned using organic solvents and stored before the fabrication of the patterned MAPs.

### Fabrication of patterned MAPs using patterned moulds

**Figure 4:**
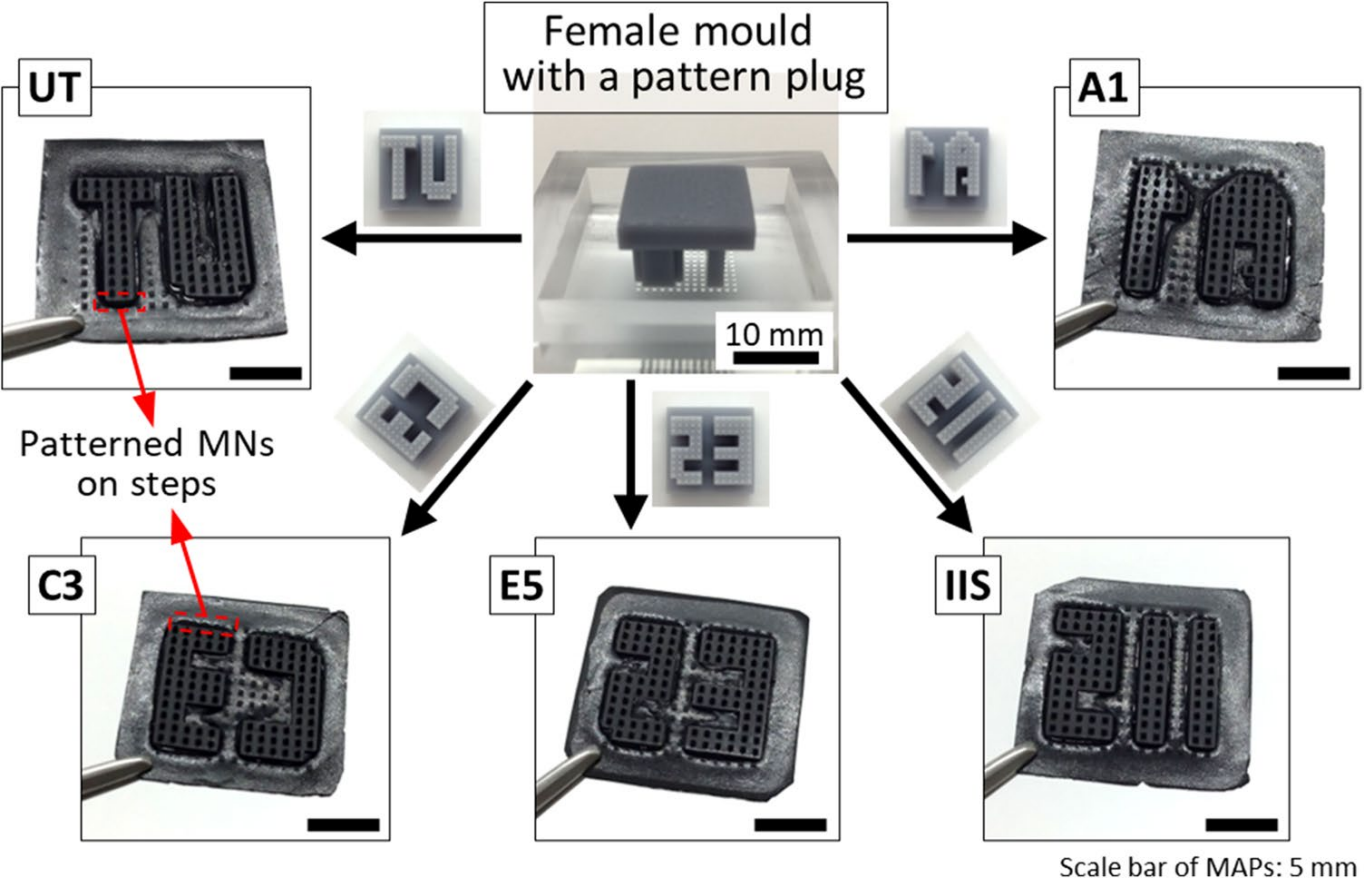
Patterned MAP fabricated by using respective patterned moulds.

Using five different patterned plugs, we successfully fabricated patterned MAPs (Fig. 4). The fabrication process was the same as that for the MAPs without patterns. The final MAPs were square-shaped with a side length of approximately 20 mm. The average thickness of the back layer was approximately 413 μm. Inside the MAPs, the pyramidal tip parts protruded with a step from the back layer because the step structures were formed in patterned moulds by the plug pillars during the filling process. The representative step structures are marked with red arrows in Fig. 4. The thickness of steps was 447.7 ± 16.6 μm among fabricated MAPs. Here, we consider step structures as having both advantages and disadvantages. Steps are expected to play the role of a pedestal for MNs, resulting in improved skin penetration during MAP application^30^. However, step structures can affect the flexibility of MAPs as they may contribute to an increase in MAP thickness. As the step structures are formed by the residue of the diluted PDMS mixture as well as pillars of patterned plugs during the filling process, we consider that the step thickness can be decreased or adjusted by controlling the amount of diluted PDMS mixture for filling.

### Animal experiments: evaluation of MAP’s dissolution during its application

Subsequently, animal experiments were performed using the fabricated patterned MAPs. Before the biotagging experiments, the application time of MAP was evaluated to determine the length of the dissolved parts and the proper time for rat experiments. Table 1 shows that the length of the dissolved MNs increases with the application time. The back layer began to dissolve after 7 min. We considered that the back layer was partially in contact with the liquefied portion of the dissolved MNs as the application time increased, resulting in the subsequent dissolution of its surface.

**Table 1:** The length of dissolved MAP with respect to different application times.

Application time	1 min	3 min	5 min	7 min	10 min
Length of dissolved part (%)	199.7 μm (24.0%)	330.8 μm (39.8%)	509.8 μm (61.4%)	783 μm (94.3%)	808.4 μm (97.3%)

Number of samples (time) = 35 (1 m), 31 (3 m), 25 (5 m), 35 (7 m), 24 (10 m).

The percentage values with the length dissolved represent how much the MNs were dissolved compared to the original lengths. Consequently, we finally chose 7 min for further animal experiments considering an entire length dissolution of over 90%.

### Animal experiments: biotagging as the direct formation of identification patterns on skin

**Figure 5:**
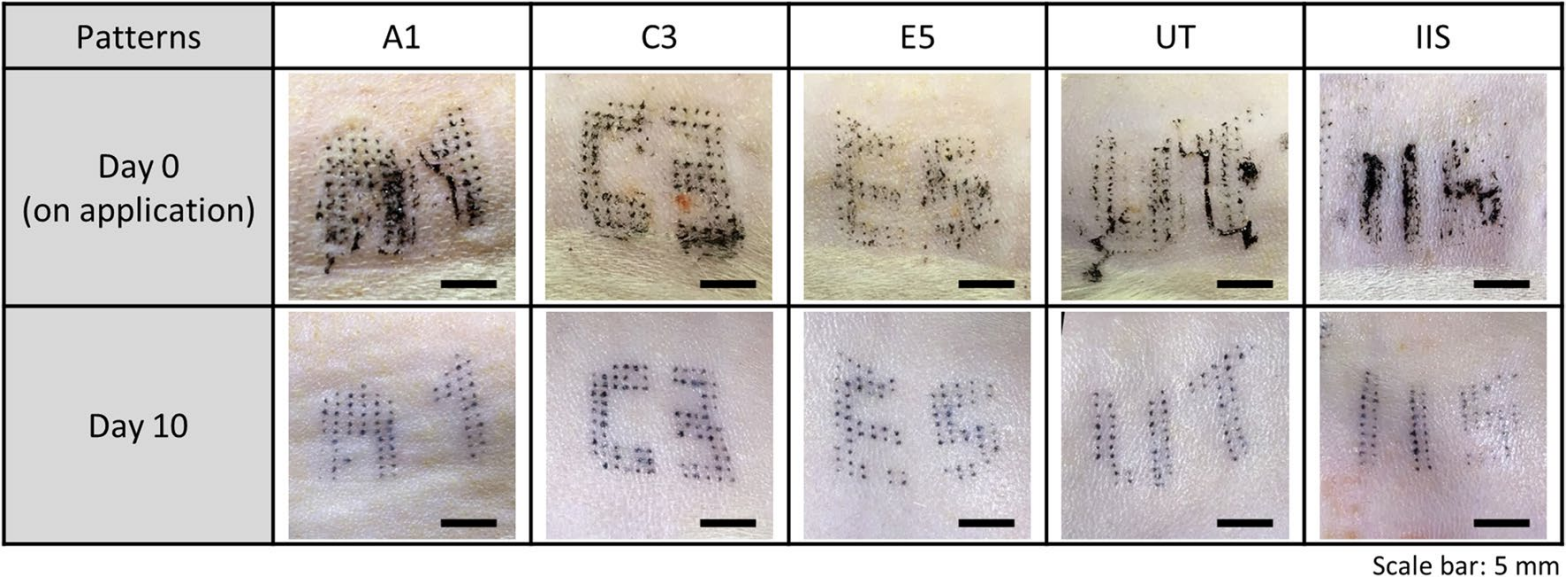
Evaluation results of biotagging using patterned MAPs on day 0 and day 10.

As a final experiment, biotagging was performed using patterned MAPs to form identification patterns on rats’ skin directly. First, we evaluated the formation of designated patterns on the skin after applying MAPs, starting on day zero. Figure 5 illustrates the pattern-formation results obtained using the patterned MAPs. In particular, the figure shows that most of the MNs in MAPs penetrated the skin, and the ink mixture was delivered inside the skin successfully. The diameters of the dots confirmed on the surface range from 240 to 400 μm. We consider the difference of diameters was caused by several factors such as the penetrated length of MNs, the amount of mixed and delivered inks during the fabrication and penetration, respectively. The minimum gap distance between two adjacent dots was 506.2 ± 55.1 μm. Although there are several factors in determining visual acuity of human naked eyes, the spatial resolution limit of human vision is generally expected to be 90–120 μm at a distance of 30–40 cm^31,32^. Overall, we expect that all patterns formed by the application of patterned MAP can be sufficiently recognised by naked eyes.

From day 0, when the MAPs were first applied, we confirmed that all the formed patterns were clearly visible to the naked eye. The results from day 10 also confirmed that all patterns remained without any loss or changes compared to those on day 0. Thus, we concluded that the ink mixture was delivered into the dermis layer by the MAP and settled in the layer. However, we observed that some dot parts were not formed as shown in A1 or E5 pattern results (Fig. 5). From the results, we confirmed that the percentage of successful dot formations was 92.1%. We consider that such pattern loss was caused by unsuccessful or insufficient penetration of MNs during MAP application, which results in the failure of ink delivery into dermis layer.

In addition, the partial dissolution of the back-layer surface was confirmed by all patterns, as described in the previous section. We confirmed that removing the residue by partial dissolution did not affect pattern formation once the ink mixture was delivered into the skin (data not shown). Concerning the loss, misalignment and distortion of character patterns, the slipping of MAPs or skin, which may occur during application, contributes to the pattern loss as well as misalignments including tilted, moved, distorted, or stretched patterns. We consider that a fixing device, such as an applicator^33^, can be helpful to fix the MAP or skin stably during its application, resulting in patterns without any shape deformations and loss.

We found that hair trimming was necessary, at least in rats, before applying MAPs to enable successful penetration into the skin. Simultaneously, the patterns were expected to be covered with hair that grew over time. We suggest that biotagging specific spots with little or no hair could be a feasible solution to hair-related issues. In addition, MAPs containing fluorescent ink that can be triggered by external stimulation, such as UV light, are considered useful for recognising patterns, even with grown hair^25^.

Second, we evaluated the long-term persistence of the patterns over one month with base MAPs and over three weeks with patterned MAPs (Supplementary Information Fig. S4). Similarly, we confirmed that all the formed patterns were retained inside the skin with clear readability. Considering the turnover time of basal cells in the epidermis layer^34^ and related literature^35^, it was expected that patterns formed by our developed MAPs would exist in the dermis layer and could play a role in identifying tags, that is, biotagging.

**Figure 6:**
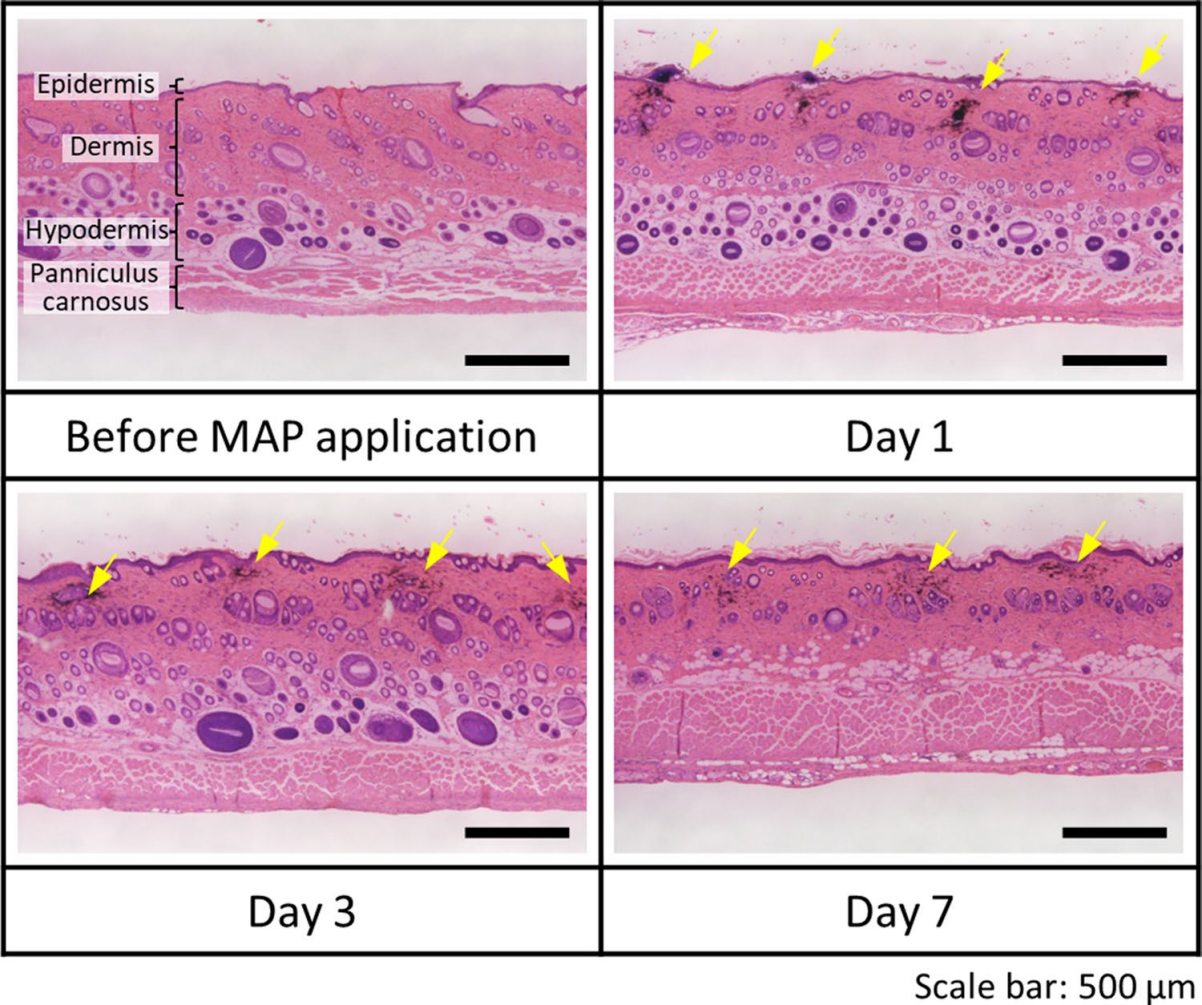
Histological analysis of dorsal skins biotagged using patterned MAPs on days 1, 3, and 7.

Finally, we harvested the dorsal skin where the MAPs were applied and performed histological analyses. We investigated two points: the first was to confirm that the ink mixture was delivered into the skin and remained inside. The second was to verify whether abnormal cell behaviour related to inflammation was observed. Figure 6 shows the histological data of the pristine dorsal skin and the MAP-applied skin on days 1, 3, and 7. We confirmed that the ink mixture was successfully delivered mainly to the dermis layers and that the ink pigments remained where delivered^36^. Compared to the skin without MAP application, black-coloured patterns with constant gaps, almost the same as the distance between MNs, were confirmed from the stained tissues (yellow arrows). Although the average length of MNs used in this research was over 800 μm, the deepest depth of ink pigment was calculated as approximately 380 μm from their difference. We considered that insufficient MNs’ penetration caused by manual application, or the flexibility of the skin contributed to these results.

From the tissue sections, the mitigation of vast neutrophils was not observed near the region where the ink mixture was delivered. Thus, acute inflammation did not occur owing to the MNs penetration and delivery of the ink mixture. However, we observed that the thickness of the epidermal layer and the number of hair follicles (circular shapes with blue colour) increased. Such morphological changes might be caused by using a depilatory cream before MAP application^37^.

## Discussion

Through overall evaluations and animal experiments, we confirmed that the developed MAPs successfully delivered the ink mixture into the skin layer and demonstrated a new biotagging method for the individual identification of animals. In this study, we proposed and demonstrated the new method to prepare customised female moulds that have various patterns using one master mould and 3D printed patterned plugs. Most of MAP-related research uses the micromoulding method that requires the female mould to fabricate MAPs. The female mould that can be prepared from the master mould, usually made of hard materials such as metals, is one important part during the fabrication process. By the way, it has a critical drawback such that we can only create one kind of the female mould with a designated pattern from one master mould. Thus, it is necessary to prepare another master mould if we want different patterns of microneedle (MN) arrays. The fabrication of the master mould with high precision is time-consuming and costly. We consider that the fabrication of female moulds can be processed fast without specific machining equipment and complex processes by using patterned plugs that can be prepared using a conventional 3D printer. Although the patterned female mould cannot be re-configurable once it is finalized, we expect the total cost would be much lower than preparing new master mould with different patterns.

Meanwhile, several challenges about the MAP application need to be addressed in future work. First, we found pattern loss or misalignment after MAP application. Especially, the pattern loss with unformed dots may hinder exact recognition of character patterns. Although we designed the patterns so that the stroke in characters forms at least 2 rows to deal with unexpected loss, it is necessary to refine character designs and to introduce additional fixing tools to achieve complete pattern formations with sufficient MNs’ penetration.

Second, animal hair can be a major issue when applying MAPs to identify individual animals. We confirmed that hair trimming was necessary to ensure the penetration of MAPs. Using MAPs with longer MNs can be a solution. However, dense hair, especially the dorsal area, could hinder their application, including MAP’s penetration. As described earlier, we expect that choosing application spots with less hair is a feasible solution. Thus, we confirmed the biotagging of the ear or tail using MAPs (Supplementary Fig. S5). Nevertheless, more consideration of pattern designs and dimensions should be provided as they have limited space for MAP applications.

For animal experiments, we used a depilatory cream to expose the skin completely for clear skin observation and MAP applications. However, we suggest that only a trimmer should be used for the skin preparation of animals to exclude relevant changes for further analyses. Compared to previous studies using a conventional syringe for tattooing, we confirmed that the delivery of tattoo ink mixtures using MAPs could alleviate various biological responses related to inflammation^38^. Therefore, we conclude that biotagging using patterned MAPs is an effective and safe method for delivering ink mixtures to the skin layer. Simultaneously, additional experiments related to evaluating the inflammation would be necessary to analyse the immunological responses due to MAP application thoroughly.

Moreover, feasible applications were considered for further applications. For example, various coloured patterns can be formed using inks of different colours. Previous studies demonstrated different coloured patterns using MAPs^25^. In addition, active pharmaceutical ingredients (API) can be mixed with multipurpose MAPs or administered simultaneously in one patch. Specific APIs controlling the fate of hair follicle cells can be integrated with patterned MAPs. Consequently, biotagging patterns can be left on the skin permanently in a hairless state.

## Conclusion

In this study, we proposed and demonstrated a biotagging method using pattern MAPs that could deliver a tattoo ink mixture and leave it in specific patterns to identify individual animals. To realise the character and number patterns on a single MAP, a new fabrication method for customised female moulds was developed and confirmed. Using animal experiments, the fabricated patterned MAPs were applied to rat skin, and long-term persistence and biotagging by the MAPs were confirmed. Based on the overall results, we conclude that the developed biotagging method using patterned MAPs is a well-established new tagging method easy to use and minimally invasive. Owing to the advantages of MAPs, the proposed method can be potentially used in various situations where animals should be handled and managed, especially in groups. Simultaneously, we expect that various applications for tagging or marking using MAPs will be expanded not only to biomedical fields but also to other research fields such as the integration with flexible electronics and the management of raw materials in manufacturing field.

## Methods

### Materials and chemicals

To fabricate the metal master mould, a tungsten carbide plate (G-KM10) was purchased (Misumi Co, Tokyo, JAPAN). A set of polydimethylsiloxane (PDMS) prepolymer and curing agent (DOWSIL™ SILPOT 184 W/C) and a dilution agent (DOWSIL™, 200 Fluid, 20cst) were used (Dow Chemical Company Midland, MI, USA) as materials for female moulds. A plant-based UV-curing resin (Elegoo, Inc., Shenzhen, CHINA), promoter, and water/oil-repellent agent (FG-5084, Fluoro Technology Co., Ltd., Aichi, JAPAN) were used for the pattern plugs and the respective female moulds. For materials of MAP, hyaluronic acid (HA) with a molecular weight of 14 kDa (Contipro a.s., Dolní Dobrouč, CZECH REPUBLIC) and tattoo ink (Carbon black colour) (World Famous Tattoo Ink, Fort Mill, SC, USA) were purchased. Tween 80 was also purchased (MP Biomedicals Inc. Santa Ana, CA, USA). For the animal experiments, an isoflurane inhalation anaesthetic (Viatris Inc., Canonsburg, PA, USA) and depilatory cream (LBS-s, Veet, Reckitt Benckiser Japan, Tokyo, JAPAN) were used.

### Design and preparation of the moulds and plugs for MAP fabrications

In this study, we used a micromoulding process with PDMS as the female mould. First, a master mould was fabricated via wire electrical discharge machining using a polished tungsten carbide plate as the substrate. The MNs were designed as a square pyramidal shape considering volume and the insertion force during penetration^39^. The length, side of the square base, and centre-to-centre of the MNs were designed to be 900, 480, and 1000 μm, respectively, and were arrayed in a square layout of 13 × 13.

In addition, two types of female moulds for MAPs were fabricated: one was a base mould without patterns. The other is the mould for the patterned MAPs. Supplementary Fig. S1 illustrates a schematic of the fabrication process. For the base mould for MAP, a mixture of PDMS prepolymer and curing agent (10:1, w/w) was poured, defoamed, and cured in an oven at 85 ℃ for 1 h. The cured female mould was released from the master mould and subsequently used to fabricate the MAP without patterns (Supplementary Information Fig. S1a).

For the female moulds for MAP with specific patterns, the pattern plugs were first designed using a 3D CAD application (Fusion 360, Autodesk, Inc., USA) and fabricated using a 3D printer (Mars 3, 4K mono LCD, Elegoo, Inc., CHINA) with UV-curable resins. The patterned plug had support pillars and pyramid-shaped tips for each pillar. Here, the dimensions of the pyramidal tips were designed to be 10% smaller than those of the base mould so that the tips of the plug could be inserted inside the cavities of the base mould. Detailed information regarding the patterns of the pattern plugs is described in the Results section. Subsequently, a hydrophobic coating was applied to the surface of the fabricated patterned plug by spraying a promoter and then a water/oil-repellent agent over twice. A mould set, including a base mould and pattern plug, was prepared by placing and fixing the plug onto the base mould. Next, a diluted PDMS mixture with the PDMS prepolymer, curing agent, and dilutant at a mixing ratio of 10:1:10 (w/w) was poured into the patterned mould set. Here, the volume of diluted PDMS mixture was adjusted so that the mixture fills not only the cavities of the base mould but the rest uniformly and sufficiently. We finally used 200 μL of diluted PDMS mixture for each mould set. Then, the mould set was vacuumed for 5 min and cured on a hotplate at 60 ℃ for 30 min. Once the mixture was cured, the plug was removed by vertical lifting (Supplementary Information Fig. S1A–C). Finally, the patterned female mould was finalised by heating the mould to 85 ℃ for 30 min inside an oven.

### Fabrication of MAPs without or with patterns

In this study, a 10% HA solution was used as the base material to fabricate all MAPs. The HA solution was prepared and mixed with the tattoo ink solution at designated ratios (HA to ink: 1, 3, 5, 7, and 15:1, v/v). All solutions were freshly prepared by mixing the two solutions and were used in each experiment.

We fabricated two types of MAPs using two different female moulds, as described in the previous section. Only the base mould was used for the MAPs without patterns (Supplementary Information Fig. S1b,c). After pouring the ink mixture into the base mould, the mixture was vacuumed and dried inside a desiccator for 24 h. Subsequently, the MAP was removed from the mould, cut, and stored in a desiccator until the experiments.

The MAP with patterns was fabricated using custom-designed female moulds prepared using the pattern plugs described in the previous section. After the patterned female moulds were prepared, patterned MAPs were repeatedly fabricated using a conventional micromoulding process (Supplementary Information Fig. S1D,E). All the MAPs were fabricated and stored under the same conditions before their use.

### Mechanical strength evaluations

The failure force of the MNs on the fabricated MAP was measured using a force measurement system consisting of a digital force gauge (ZTA-5N, IMADA Co., Ltd., Japan) and a test stand (MX2-500N, IMADA Co., Ltd., Japan). The compressive force with respect to the displacement was measured by pressing a single microneedle vertically at a rate of 1.5 mm/min using a pin attachment with a diameter of 1 mm. The compression force and displacement were continuously measured until the tip of the MN completely collapsed, and the failure force was recorded as the force suddenly decreasing upon the failure of the MN structure.

### Biotagging evaluation using patterned MAPs with animal experiments

Five-week-old female Sprague-Dawley rats (Jcl:SD) were purchased (CLEA Japan, Inc., Tokyo, JAPAN) for in vivo tests. All rats were grouped and housed at cages inside a ventilated rack under controlled environment (22–23 ℃, 40–60% RH, 12 h light–dark cycle). All experiments were performed on 6–8 weeks old rats after one week of adaptation to the environment. The rats were first placed inside a chamber filled with 4% vaporised isoflurane at a flow rate of 2 L/min using an animal inhalation anaesthesia machine (WP-SAA01, LMS Co., Ltd., Tokyo, JAPAN). After the induction of inhalation anaesthesia, the rats were moved onto a heating plate to maintain body temperature while being masked to maintain anaesthesia. During the experiments, the isoflurane concentration was maintained between 1 and 2% at a flow rate of 2 L/min. The hair on the dorsal skin was cut using a pet hair trimmer, and a depilatory cream was subsequently applied to expose the hairless skin. The amount and application time of the cream was determined as 0.1 g/cm^2^ and 90 s, respectively. After depilation, the cream was thoroughly wiped off with dried and wet wipes. MAPs were applied to the skin using a thin metal plate on the opposite side as a support to avoid direct physical damage to the rat body^21^. During the application, MAPs were first pressed down at around 11 N for 5 s and then maintained at a pressure of around 4 N for designated time. Regarding the evaluation of MAP dissolution, the time of MAP application was evaluated and determined by measuring the respective dissolved lengths of the MNs with changing application times. The skin was photographed after MAP application at each evaluation. All animal experiments were reviewed and approved by the Research Ethics Committee of the Institute of Industrial Science, The University of Tokyo (ethics approval number: 04-05 in 2022). All experiments and related methods were performed in accordance with the ARRIVE guidelines as well as University of Tokyo Animal experiment implementation regulations.

### Histological analysis

For histopathology, the full thickness of the dorsal skin, where MAPs were applied, was harvested from the experimental groups on days 1, 3, and 7 after the animals in each group were euthanised by carbon-dioxide inhalation. Harvested specimens were fixed in 4% paraformaldehyde, dehydrated, and embedded in paraffin. The paraffin-embedded tissue blocks were cut into slices with a thickness of 5 μm and 100 μm gaps between slices. Haematoxylin and eosin (H&E) staining was performed according to routine protocols to stain and visualise the constituents of cells and tissues. The images were captured using a digital microscope (VH-5500, Keyence Co., Osaka, JAPAN).

### Supplementary Information

Supplementary Figures.

## Data Availability

The data that support the findings of this study are available from the corresponding author upon reasonable request.
