# Supplementary material for: Biotagging method for animal identification using dissolvable microneedle arrays prepared by customisable moulds
Source: Sci Rep. 2023 Dec 21;13:22843. doi: 10.1038/s41598-023-50343-6 (PMC10739709; doi:10.1038/s41598-023-50343-6)
Supplement: Supplementary file 1 — Supplementary Figures. [file 41598_2023_50343_MOESM1_ESM.docx]

**Supplementary Information**

**Biotagging method for animal identification using dissolvable microneedle arrays prepared by customisable moulds**

Jongho Park and Beomjoon Kim


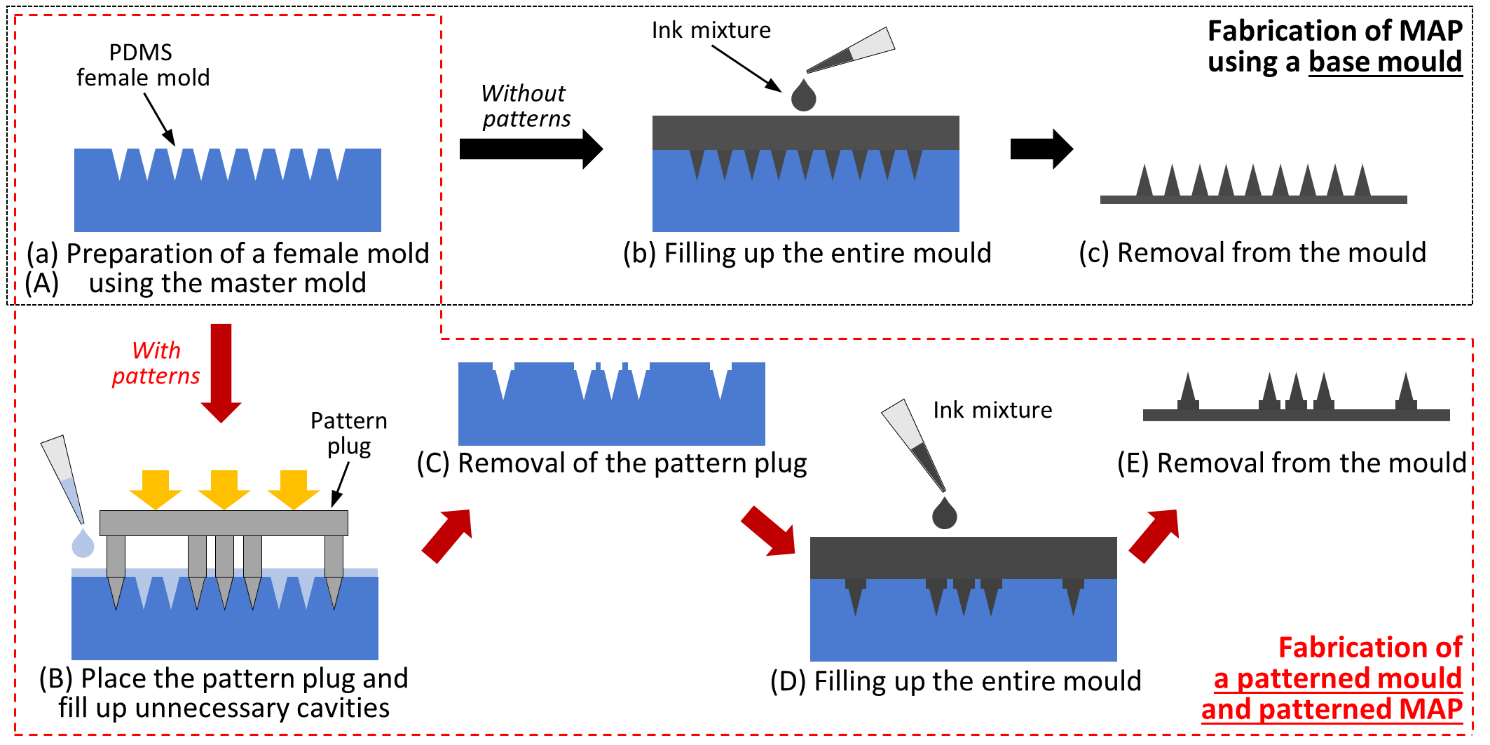


**Figure S1 Schematic diagram of the fabrication processes for a base mould and a patterned mould as well as MAP.**


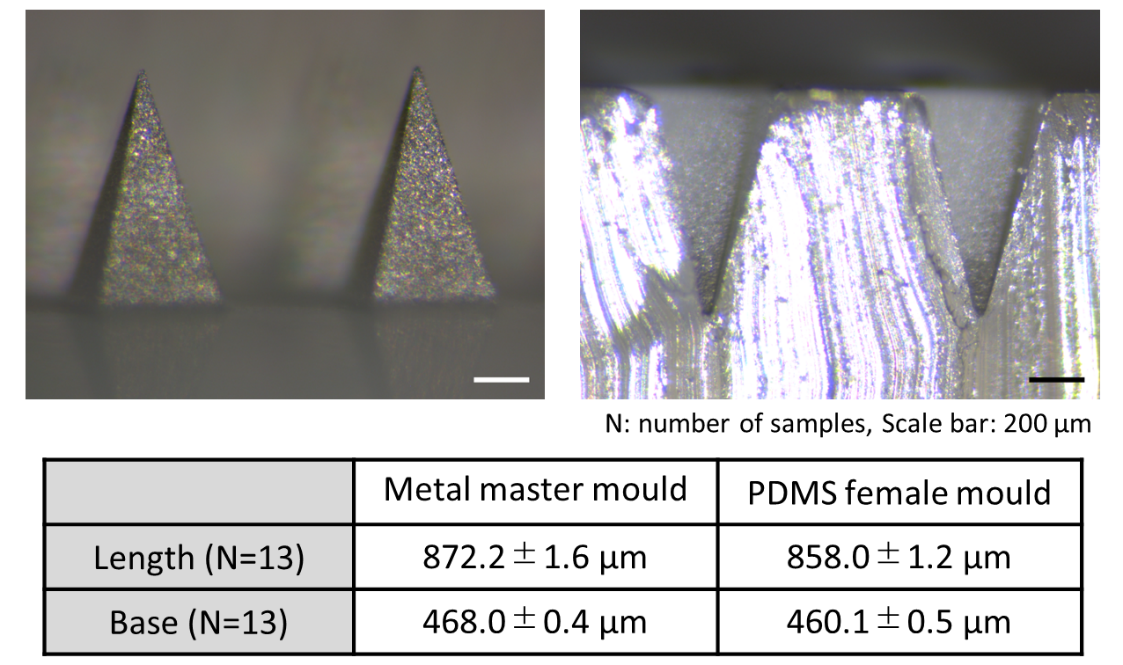


**Figure S2 Representative microscopic images and dimensions of the metal master mould as well as a PDMS female mould.**


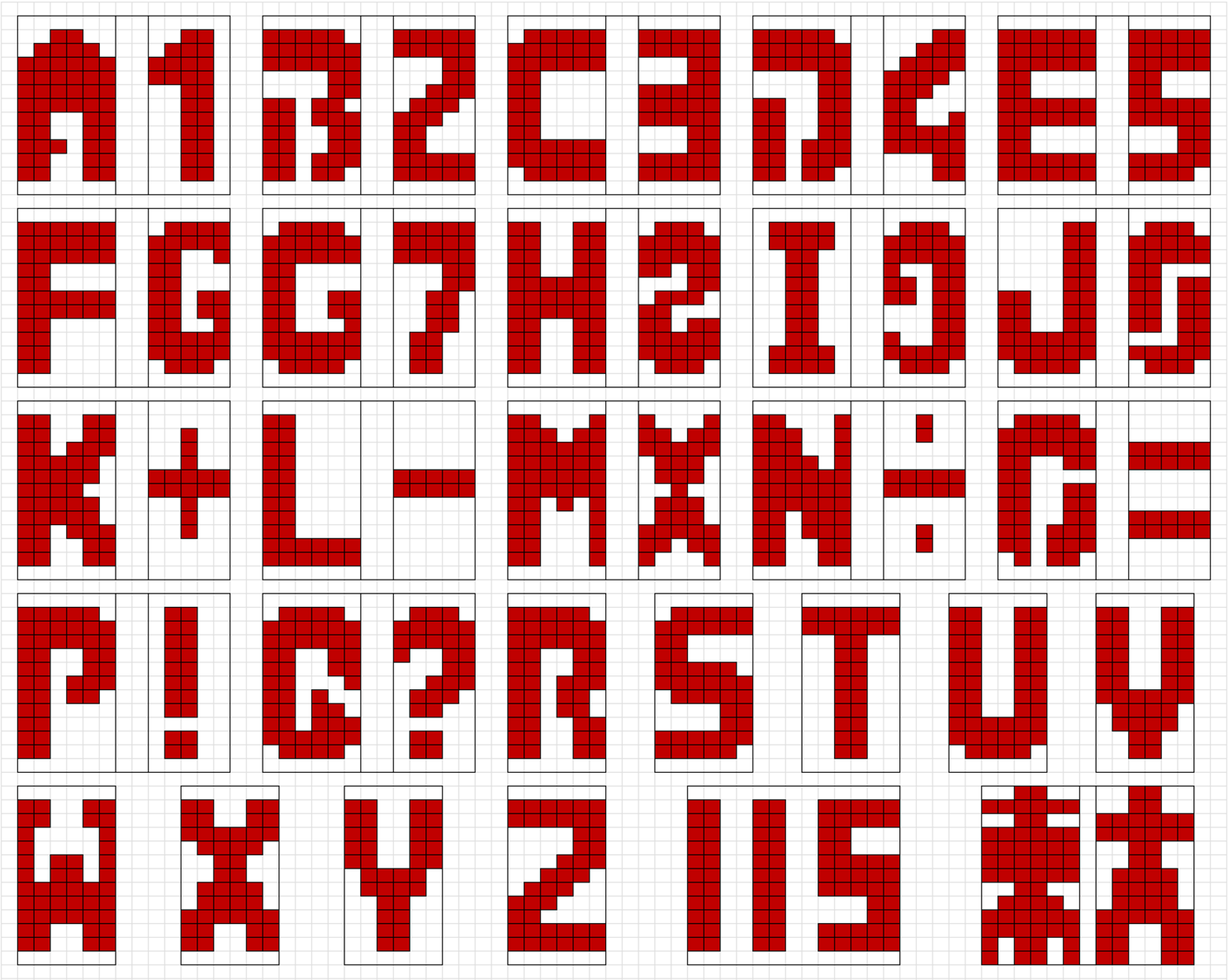


**Figure S3 Matrix array design for patterns of English alphabet characters, numbers, and symbols that were used for pattern plugs.**


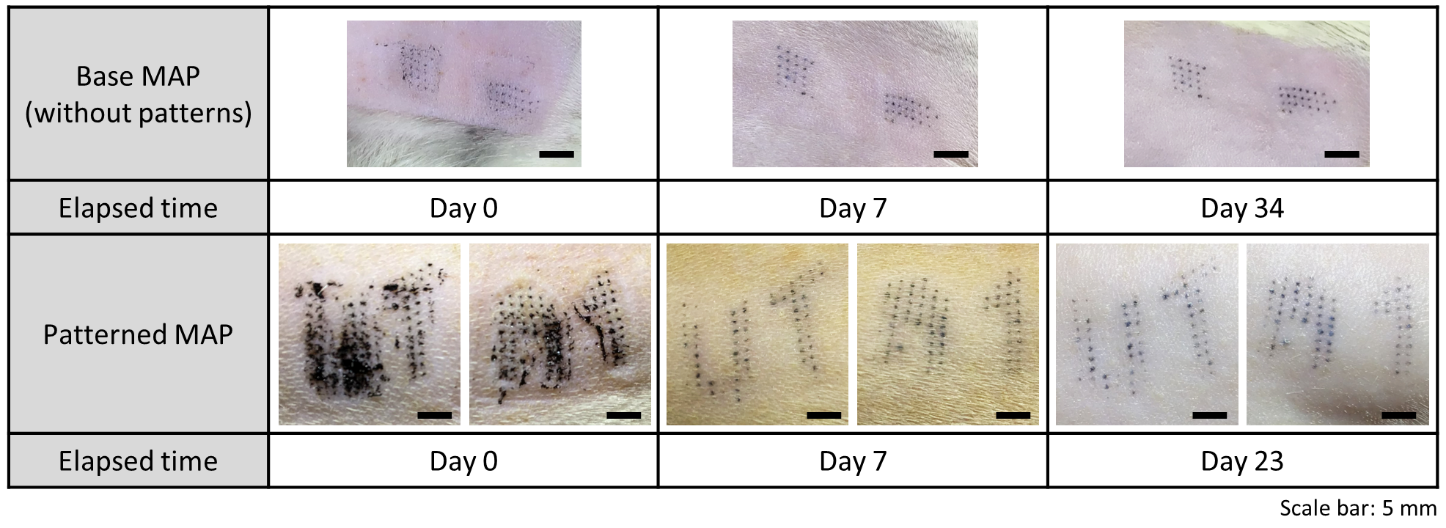


**Figure S4 Long-term evaluation of patterns formed by base MAPs and patterned MAPs.**


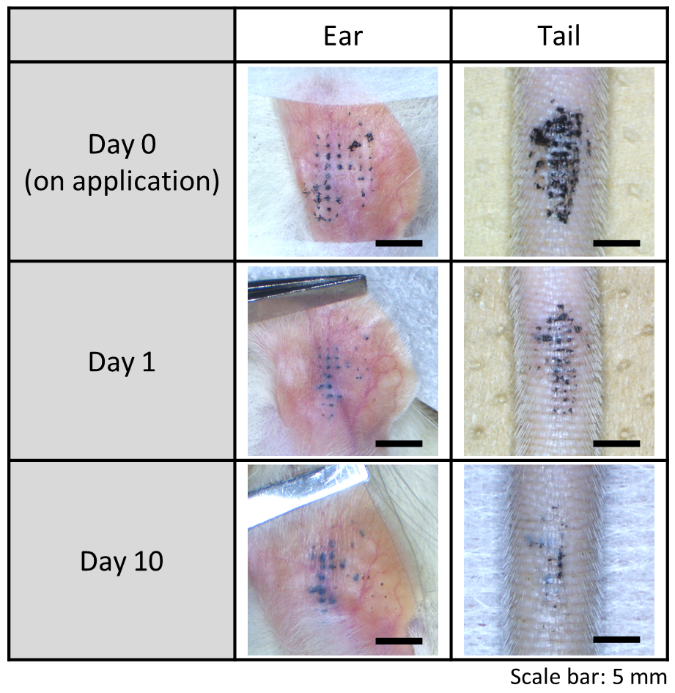


**Figure S5 Biotagging results using developed MAPs on the ear and tail that are representative spots with less hair.**
